# Uncovering the Mechanisms of Chinese Herbal Medicine (MaZiRenWan) for Functional Constipation by Focused Network Pharmacology Approach

**DOI:** 10.3389/fphar.2018.00270

**Published:** 2018-03-26

**Authors:** Tao Huang, Ziwan Ning, Dongdong Hu, Man Zhang, Ling Zhao, Chengyuan Lin, Linda L. D. Zhong, Zhijun Yang, Hongxi Xu, Zhaoxiang Bian

**Affiliations:** ^1^Lab of Brain and Gut Research, School of Chinese Medicine, Hong Kong Baptist University, Kowloon Tong, Hong Kong; ^2^School of Pharmacy, Shanghai University of Traditional Chinese Medicine, Shanghai, China; ^3^Engineering Research Center of Shanghai Colleges for TCM New Drug Discovery, Shanghai, China; ^4^Guangzhou Research Institute of Snake Venom, Guangzhou Medical University, Guangzhou, China; ^5^YMU-HKBU Joint Laboratory of Traditional Natural Medicine, Yunnan Minzu University, Kunming, China; ^6^Hong Kong Chinese Medicine Clinical Study Centre, Hong Kong Baptist University, Kowloon Tong, Hong Kong

**Keywords:** MaZiRenWan, component group, functional constipation, focused network pharmacology, representative compound, Chinese Herbal Medicine

## Abstract

MaZiRenWan (MZRW, also known as Hemp Seed Pill) is a Chinese Herbal Medicine which has been demonstrated to safely and effectively alleviate functional constipation (FC) in a randomized, placebo-controlled clinical study with 120 subjects. However, the underlying pharmacological actions of MZRW for FC, are still largely unknown. We systematically analyzed the bioactive compounds of MZRW and mechanism-of-action biological targets through a novel approach called “focused network pharmacology.” Among the 97 compounds identified by UPLC-QTOF-MS/MS in MZRW extract, 34 were found in rat plasma, while 10 were found in rat feces. Hierarchical clustering analysis suggest that these compounds can be classified into component groups, in which compounds are highly similar to each other and most of them are from the same herb. Emodin, amygdalin, albiflorin, honokiol, and naringin were selected as representative compounds of corresponding component groups. All of them were shown to induce spontaneous contractions of rat colonic smooth muscle *in vitro*. Network analysis revealed that biological targets in acetylcholine-, estrogen-, prostaglandin-, cannabinoid-, and purine signaling pathways are able to explain the prokinetic effects of representative compounds and corresponding component groups. In conclusion, MZRW active components enhance colonic motility, possibly by acting on multiple targets and pathways.

## Introduction

Functional constipation (FC) is a common gastrointestinal (GI) disorder characterized by slow bowel movement and defecation difficulties. It influences over 14.0% adults worldwide (Suares and Ford, 2011). Female gender, old age, and low socioeconomic status are risk factors for people to have FC (Suares and Ford, 2011). FC cause worse life quality and significant financial burden of whole society (Liem et al., 2009; Peery et al., 2012, 2015). People with mild or moderate FC can be treated with high-fiber or laxatives (Rao et al., 2015; Christodoulides et al., 2016), while patients with severe FC need special cares and aggressive therapies. Several pharmacological therapeutics have been approved for FC, including diphenyl mechanics or derivatives, anthraquinone, 5-hydroxytryptamine receptor 4 (5-HT_4_) agonist, chloride channel type 2 activator, guanylate cyclase C receptor agonist, apical sodium bile acid inhibitors (Nelson et al., 2016). More than 50% FC patients are not completely satisfied with current therapies (Nelson et al., 2016), and alternative therapies for FC are required.

MaZiRenWan (MZRW, also known as Hemp Seed Pill) is an herbal formula for constipation from Traditional Chinese Medicine (TCM). It was firstly recorded in a TCM classic book, *Discussion of Cold-Induced Disorders* (*Shang Han Lun*) (Jing, 1997; Mitchell et al., 1998), in about 2,000 years ago. MZRW is comprised of six herbs, including *Fructus cannabis* (*Huo Ma Ren*, HMR), *Radix et rhizoma rhei* (*Da Huang*, DH), *Semen Armeniacae Amarum* (*Ku Xing Ren*, KXR), *Radix paeoniae Alba* (*Bai Shao*, BS), *Cortex magnolia officinalis* (*Hou Pu*, HP), and *Fructus aurantii immaturus* (*Zhi Shi*, ZS) (Cheng et al., 2011).

A systematic review of the published literature showed that MZRW is the most frequently used TCM formula for constipation (Zhong et al., 2016), but there is little strict clinical evidence to prove its efficacy. In the randomized, placebo-controlled clinical study with 120 FC patients, we demonstrated that MZRW is significantly better than placebo in improvement of bowel movement during the drug treatment period, and such effect is more sustainable than placebo during the 8 weeks follow-up period (Cheng et al., 2011). We also identified 10 major compounds from MZRW in rat plasma by UPLC-MS/MS (Hu et al., 2015) to facilitate the pharmacokinetic study of MZRW in healthy volunteers (Hu et al., 2017). However, the active components and the mechanism-of-actions by which MZRW utilized to alleviate FC, are still unclear.

In this study, we investigated the pharmacological actions of MZRW for FC with a novel idea: focused network pharmacology. In the classic network pharmacology studies for TCM, hundreds of herbal compounds were retrieved from database and/or literatures, and hundreds/thousands of targets were mapped (Zeng et al., 2017; Chen et al., 2018). The network comprised of huge number of compounds and targets could explain the holistic and complex effects of TCM, but they will also result in numerous mechanistic hypotheses to be tested. To confer such defects, we only included the compounds validated by quantitative methods, then further focused on the representative compounds with chemical space analysis.

To precisely define the effective compounds, we used UPLC-QTOF-MS/MS to identify compounds in MZRW extract (after preparation), and compounds in biological samples of rats after oral administration of MZRW. Followed-by computational analysis, we found that the identified compounds can be clustered as “component groups” based on pairwise chemical similarity. Five representative compounds for the component groups were selected and their effects on rat intestinal tract contractions were tested. The relevant disease targets and signaling pathways were investigated.

## Materials and Methods

### Compounds Identification

#### Reagents and Materials

All the six herbal medicines (HMR: RM130121-08; DH: 130524; BS: RM130128-11; KXR: RM121026-04; HP: RM121026-05; ZS: RM121210-11) contained in MZRW were supported and authenticated by PuraPharm Company, Nanning department (Nanning, China). The reference standards of gallic acid, amygdalin, paeoniflorin, hesperidin, neohesperidin, naringin, magnolol, honokiol, aloe emodin, physcion, chrysophanol were purchased from Shanghai Yuan-Ye Bio-technology company (Shanghai, China). Emodin, rhein, albiflorin were purchased from the National Institute for the Control of Pharmaceutical and Biological Products (Beijing, China). The purity of all these reference standards was ≥98.0% (HPLC). HPLC grade of acetonitrile, methanol and formic acid were purchased from Merck (Darmstadt, Germany). Deionized water was purified by the Millipore water purification system (Millipore, Milford, MA, United States). All other reagents used were of analytical grade. Twelve male Sprague-Dawley rats (220–250 g) were kindly provided by the Experimental Animal Center of Chinese University of Hong Kong (Hong Kong). The rats were bred in an environmentally controlled room (22 ± 2°C, relative humidity 50 ± 20%) with a natural light-dark cycle for 7 days before the experiment carried out. The animal study was carried out in accordance with the Guideline for Animal Experimentation of Hong Kong.

#### Instrumentation and Analytical Conditions

Liquid Chromatographic analysis was performed on an Agilent 1290 UPLC system, consisting of a 1290 binary pump solvent management system, an 1290 TCC, and an 1290 auto-sampler. A waters Acquity BEH C_18_ column (100 mm × 2.1 mm, 1.7 μm) was employed and the column temperature was maintained at 40°C. The mobile phase was composed of A (0.1% formic acid in water) and B (0.1% formic acid in acetonitrile) using a gradient elution of 2–5% B at 0–2.5 min, 5–35% B at 2.5–10 min, 35–75% B at 10–14 min, 75–100% B at 14–16 min, 100% B at 16–20 min, 100–2% B at 20–20.1 min and 2% B at 20.1–24 min with a flow rate set at 0.40 mL/min. The auto-sampler was conditioned at 4°C and the injection volume was 3 μL.

Mass detections were performed using an Agilent 6540 Quadruple-Time of flight mass spectrometer (6540 Q-TOF-MS, Agilent Technologies, Santa Clara, CA, United States) equipped with an AJS electrospray ionization source (ESI). The ESI source was set in negative ionization mode. The parameters in the source were set as follows: Gas temperature, 300°C; gas flow, 8.0 L/min; nebulizer, 45 psi; sheath gas temperature, 350°C; sheath gas flow, 8.0 L/min; capillary voltage, 3000 V; nozzle voltage, 500 V; fragmentor voltage, 150 V; Oct RFV, 600 V; and skimmer voltage 65.0 V. charging, 3000 V; The operations, acquisition, and data analysis were operated under MassHunter Acquisition Software Version B.06.00 (Agilent Technologies). Accurate mass of all mass peaks were measured and recorded with a full scan mode set at a mass range from m/z 100 to 1700. The molecular masses of the precursor and product ions were accurately determined. Reference masses 966.00072500 and 112.98558700 in negative ionization mode.

To optimize signals and obtain maximal structural information, the Q-TOF MS/MS scan was also conducted. The energies for collision-induced dissociation (CID) were set at two fixed collision energies of 15 and 35 V, other Q-TOF fragmentation parameters were the same as those for Q-TOF MS, and data were also acquired over a mass range from m/z 100 to 1700.

#### Preparation of MZRW Herbal Extract for Administration

Six pulverized herbal ingredients, *Fructus Cannabis* (892.9 g), *Semen Armeniacae Amarum* (446.4 g), *Radix et Rhizoma Rhei* (446.4 g), *Fructus Aurantii Immaturus* (223.2 g), *Cortex Magnoliae Officinalis* (267.9 g) and *Radix Paeoniae Alba* (223.2 g) were mixed together and decocted with 8-flood volumes of water (1:8, w/v) for 2.5 h, then the decoction was filtered. The filtered decoction was concentrated by rotary evaporation under vacuum at 55°C. The residue was then freeze dried. Finally, the dried residue was dissolved in water to obtain MZRW oral solution with a concentration of 0.5 g/mL. Raw materials were stored at room temperature. MZRW preparations were stored at -20°C.

#### *In Vivo* Study

Twelve male Sprague-Dawley (SD) rats, weighing 250–300 g, were fasted for 12 h with free access to water before experiments. The rats were randomly divided into two groups. One group was for plasma collection, and the other group for feces sample collection. Blood and feces samples before administration of each rat were collected for self-control study. Single dosage of MZRW oral solution was orally administered to each rat at a dose of 1g/100 g bodyweight. Then blood samples of about 500 μL were collected at 15 min, 30 min, and 3 h after administration by cutting tail. Feces samples were collected in 12 h after administration by metabolic cages. The blood samples were then centrifuged at 5000 rcf for 5 min to obtain plasma samples, which were frozen immediately and stored at -20°C until analysis; meanwhile, the feces sample were directly stored at -20°C until analysis.

#### Sample Preparation

MaZiRenWan dried extract was accurately weighted and dissolved in 70% methanol-water (30 mg/mL, w/v) following with ultrasonic extraction at room temperature for 30 min. Then the extract solution was centrifuged at 13000 rcf for 10 min. The supernatant was injected for components identification of MZRW.

Reference compounds of gallic acid, amygdalin, paeoniflorin, hesperidin, neohesperidin, naringin, magnolol, honokiol, aloe emodin, physcion, chrysophanol, emodin, albiflorin, and rhein were accurately weighted and dissolved in methanol to give a stock solution of 200 μg/ml. Then mixed standard solution of this 14 compounds at 5 μg/ml were prepared for the confirmation of compounds in MZRW.

Rat plasma sample (300 μL) was added to a 1.5 mL tube (Eppendorf). Then extraction was performed by adding 700 μL methanol to precipitate protein and extract components from the plasma. The sample was vortexed for 2 min, and then centrifuged for 10 min at 13000 rcf. The supernatant was transferred into another tube and dried under a flow of nitrogen gas. The residue was re-constituted in 100 μL methanol solutions, and centrifuged (13000 rcf for 10 min). The supernatant was transferred to an auto-sampler vial, 3 μL was injected into the UPLC-MS/MS system for analysis.

Feces samples were accurately weighted and mixed with normal saline as 1g/2ml. Then ultrasonic extraction (30 min at room temperature) was conducted to extract the components in feces. The mixed solution was then centrifuged. Three microliter supernatant was injected for LC-MS/MS analysis.

### Chemical Space Analysis

The Venn’s diagram was drawn by online tool (Venny 2.1^1^) (Oliveros, 2007–2015). Fingerprint similarity between compounds was calculated with ECFP-4-like Morgan (radius = 2) (Rogers and Hahn, 2010) in RDKit^2^. The clustering map was made with *seaborn* (version 0.8.0) package in Python. The PCA analysis was performed with *scikit-learn* (Pedregosa et al., 2011) package in Python.

### Organ Bath

Adult Sprague-Dawley male rats (250–300 g) were obtained from Guangdong Medical Laboratory Animal Centre. Rats were kept on a 12 h light/dark cycle at 23 ± 2°C with free access to food and water. Experimental protocols were approved by the Committee on the Use of Human and Animal Subjects in Teaching and Research, Hong Kong Baptist University (HASC/16-17/0331). Emodin, Naringin, Amygdalin, Albiflorin, and Honokiol were purchased from Shenzhen ChemStrong Scientific Co., Ltd. The purity of the drugs were equal or above 99%. The drugs were dissolved in DMSO and the final concentration of DMSO in solution were limited in 1‰.

The rats were euthanized with CO_2_, and a 4-cm long segment of distal colon was dissected out. The content of the colonic segment was flushed with Krebs solution and then cut into 4 segments, each 1 cm in length. Each segment was mounted longitudinally and the contraction of longitudinal smooth muscle was recorded. Briefly, the lower end of the colonic segment was tied with a holder at the bottom of the organ bath, while the upper end was connected to an isometric force transducer with a silk thread (Braided silk wax, US 5/0, Pearsalls Ltd., United Kingdom) for recording mechanical activity. Colonic contractions were recorded using the PowerLab system and Chart5 software (AD instrument Ltd., Australia). The segment was bathed in an oxygenated (95%O_2_–5% CO_2_) Krebs solution at 37°C. Krebs solution contained (in mM) 119 NaCl, 4.5 KCl, 1.2 MgCl_2_, 25 NaHCO_3_, 1.2 KH_2_PO_4_, 2.5 CaCl_2_, and 11.1 Glucose. The colonic segments were allowed to equilibrate for 1 h before the experiment started. During the equilibration period, the segment was rinsed every 20 min with Krebs solution and the basal tension was maintained (Lin et al., 2015).

The amplitude (in g) of contractions was measured and expressed as active tension (force/area, g/mm^2^), using the following equation: force/area = grams tension/ [gram wet wt/ (1.05 × L_0_)], with 1.05 as the density of smooth muscle (Bossone et al., 2001). The optimal length (*L*_o_) was obtained using the similar method described before.

### Network Analysis of Representative Compounds

#### Referenced Targets Analysis

For each of the representative compounds of MZRW, the corresponding biological targets were searched from BindingDB^3^ (Chen et al., 2001; Liu et al., 2007; Gilson et al., 2016). These reported targets were manually checked and curated by searching relevant references from PubMed. Full-text of publications were retrieved and read by one expert. If no relevant publications were founds with that target, it was removed from the referenced targets list. If relevant publications were found, the associated bioactivity data were also gathered.

#### Predicted Targets Analysis

The targets of MZRW representative compounds were predicted by the MOST method (Huang et al., 2017). Based on the bioactivity type, the pK_i_, pIC_50_, and pEC_50_ datasets were generated from the CHEMBL20 database (Gaulton et al., 2012) by using procedures described in previous work (Huang et al., 2017). The machine learning model was training with dataset from CHEMBL19 and the performance of prediction were summarized in Supplementary Table S8. For each compound, the SMILES string was used for dataset searching. The results were filtered by the following criterions: *T*c ≥ 0.45 and *p* < 0.05.

#### Target-Constipation Link Analysis

For all the referenced and predicted targets, their links with constipation were validated by searching PubMed^4^. The following key words were used: “[target gene symbol or full name] AND constipation.” Only targets with constipation-relevant publications were remained.

## Results

### Identification of Compounds in MZRW Extract, and in Rat Plasma and Feces After Oral Administration of MZRW

**FIGURE 1:**
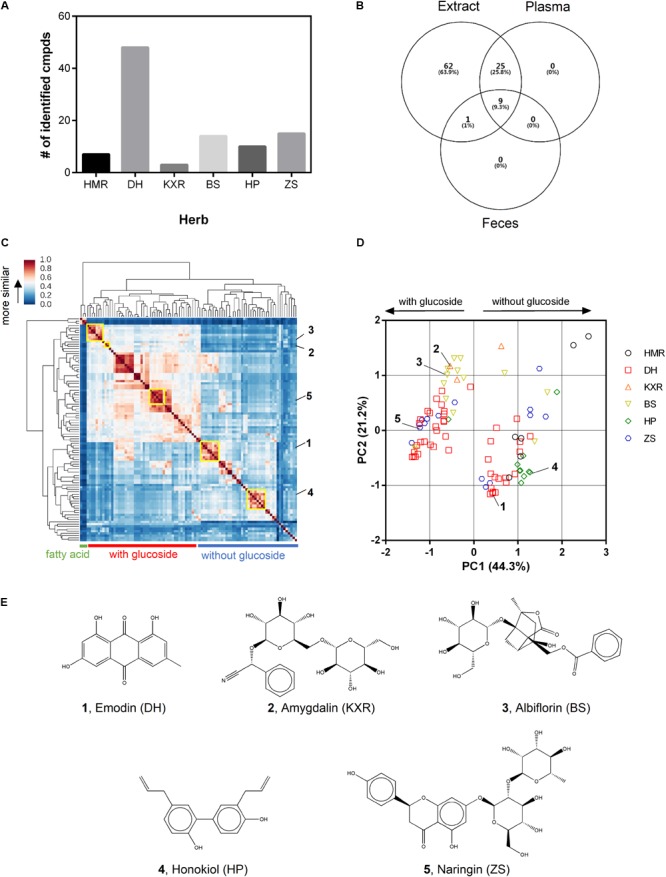
Identification of compounds of MZRW in extract and biological samples and selection of representative compounds. **(A)** Histogram of number of identified compounds by herbs. **(B)** Venn diagram of number of identified compounds by extract and biological samples. **(C)** Clustering analysis of identified compounds based on chemical similarity. Component groups were highlighted by yellow rectangle. **(D)** PCA analysis of identified compounds according to chemical similarity. **(E)** Representative compounds of each component group.

We set out to identify compounds in the MZRW extract, as well as in plasma and feces samples of rats orally administrated with MZRW extract. In total, 97 compounds were identified by UPLC-QTOF-MS/MS in MZRW extract (Supplementary Table S1). Among them, 7 were from HMR, 48 were from DH, 3 were from KXR, 14 were from BS, 10 were from HP, and 15 were from ZS (**Figure 1A** and Supplementary Table S1). We also identified the compounds in rat plasma and feces samples after oral administration: 34 compounds can be found in rat plasma, and 10 can be found in rat feces (**Figure 1B**). Nine compounds were commonly identified in rat plasma and feces, while only 1 (naringenin) was found in feces (**Figure 1B**).

Next we analyzed the chemical spaces of the identified 97 compounds. By calculating the fingerprint similarity and analyzing the clusters, these compounds were classified into hierarchical groups (**Figure 1C**). In general, they are fatty acids and non-fatty acid compounds. The fatty acids, including linoleic acid, oleic acid, and sativic acid, are oil components from HMR. The non-fatty acid compounds can be further categorized into compounds with glucoside (e.g., albiflorin), and without glucoside (e.g., emodin) (**Figures 1C,D**). Very interestingly, these compounds were clustered into small groups based on chemical similarity. Within such group, the compounds are highly similar to each other in terms of chemical structure, and most of them are from the same herb (Supplementary Tables S2–S6). It is well-known that similar compounds are likely to have similar bioactivity profiles (Bender and Glen, 2004), the clustered compounds suggest a way to abstract the complex chemical space of MZRW. We use the concept of “component group” to define the clustered compounds. Firstly, a component group contain compounds with highly similar structures, and most of the compounds are from the same herb. Secondly, a component group can be treated as a whole in investigation of pharmacological actions. Thirdly, the biological function of a component group can be demonstrated by a representative compound, which is abundant in herb, in formula extract, and in biological samples.

With these definitions, we were able to locate five component groups, which are from DH, KXR, BS, HP, and ZS (**Figure 1C**), respectively (Supplementary Tables S2–S6). Five representative compounds were selected because of their high abundance in MZRW (Hu et al., 2015), commercial availability, and frequent use in pharmacology study: emodin for DH, amygdalin for KXR, albiflorin for BS, honokiol for HP, and naringin for ZS (**Figure 1E**). These representative compounds were used with organ bath experiments to elucidate the pharmacological actions of MZRW. The component group from HMR were excluded, because they are fatty acids which are hard to be tested in organ bath.

### Effects of the Representative Compounds of MZRW on the Contractions of Rat Colonic Segments

A major part of FC patients are with symptom of slow bowel movement, which can be alleviated by MZRW treatment. Thus, we tested if the representative compounds could induce contractions of the rat colonic segments in organ bath experiments.

**FIGURE 2:**
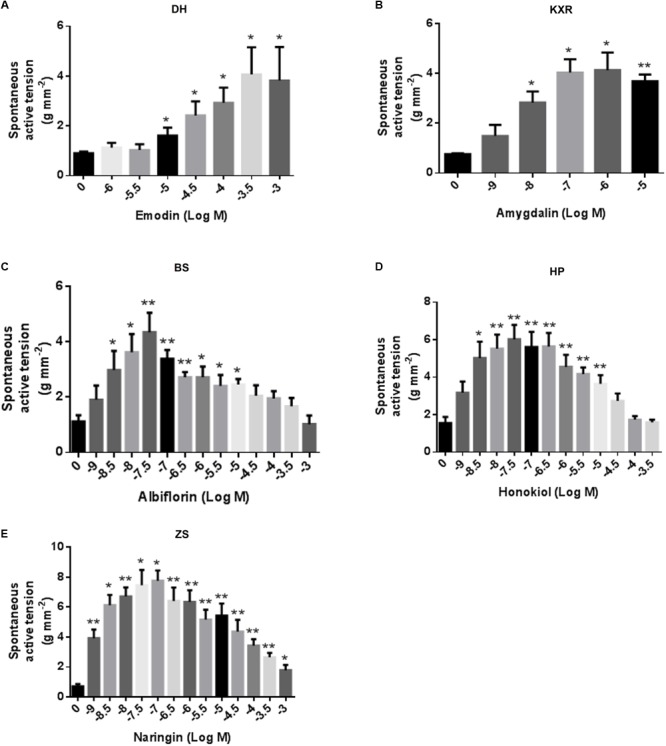
Effects of MZRW representative compounds on spontaneous contraction of colonic smooth muscle in rats *in vitro*. Effects of emodin **(A)**, amygdalin **(B)**, albiflorin **(C)**, honokiol **(D)**, and naringin **(E)** on spontaneous contraction of colonic smooth muscle. Data are mean ± SEM (*n* = 4–6). Significant difference is indicated by ^∗^*P* < 0.05 and ^∗∗^*P* < 0.01 compared with control.

Emodin (1 μM to 0.3 mM) concentration-dependently increased colonic smooth muscle contractions (**Figure 2A**). Amygdalin (1 nM to 1 μM) concentration-dependently increased colonic smooth muscle contractions (**Figure 2B**). Albiflorin (1–30 μM) concentration-dependently increased colonic smooth muscle contractions while decreased the contraction at the dose range from 100 μM to 1 mM (**Figure 2C**). Honokiol (1–30 nM) concentration-dependently increased colonic smooth muscle contractions while decreased the contraction at the dose range from 300 nM to 100 μM (**Figure 2D**). Naringin (1–100 nM) concentration-dependently increased colonic smooth muscle contractions while decreased the contraction at the dose range from 1 μM to 1 mM (**Figure 2E**).

These results suggested that all the five representative compounds have prokinetic effects *in vitro*. Next we asked, what’s the biological targets mediating such prokinetic effect?

### Representative Compound-Target-Disorder Network of MZRW for FC

We used two complementary methods to investigate the constipation-relevant targets of representative compounds.

Firstly, the targets of representative compounds were retrieved from the BindingDB (see footnote 3) (Chen et al., 2001; Liu et al., 2007; Gilson et al., 2016), and curated manually, resulting in the “referenced targets” list (Supplementary Table S7). No referenced targets can be found with amygdalin and albiflorin, while 7 targets for emodin [ESR1 (Matsuda et al., 2001), ESR2 (Matsuda et al., 2001), CSNK2A1 (Sarno et al., 2003), EPHX2 (Lee et al., 2015), PTP4A3 (Han et al., 2012), LCK (Chang and Geahlen, 1992), and ELANE (Zembower et al., 1992)], 7 targets for honokiol [PTGS1 (Schuhly et al., 2009), PTGS2 (Schuhly et al., 2009), ALOX5 (Schuhly et al., 2009), CNR1 (Rempel et al., 2013), CNR2 (Rempel et al., 2013), GABA_A_ (Taferner et al., 2011), and RXR (Kotani et al., 2010)], and 1 target for naringin [CYP19A1 (Endringer et al., 2008)] were found (**Table 1**).

**Table 1:** Referenced and predicted targets of MZRW representative compounds.

Herb	Compound	Referenced targets^a^	Predicted targets^b^
DH	Emodin	ESR1 (Matsuda et al., 2001); ESR2 (Matsuda et al., 2001); CSNK2A1 (Sarno et al., 2003); EPHX2 (Lee et al., 2015); PTP4A3 (Han et al., 2012); LCK (Chang and Geahlen, 1992); ELANE (Zembower et al., 1992)	ESR2;ESR1;SRC;PIM1;IGF1R;PPARG;CYP1B1;MET;AKR1B1; ALOX5;FLT3;CSNK2A1;AXL;TNKS2
KXR	Amygdalin	None	SLC5A4;ACHE;SLC5A1;P2RY14;SLC5A2;CA14;CA12;CA2;CA9;CA1
BS	Albiflorin	None	SLC5A4;SLC5A1;SLC5A2;CA12;CA2;CA14;CA9
HP	Honokiol	PTGS1 (Schuhly et al., 2009); PTGS2 (Schuhly et al., 2009); ALOX5 (Schuhly et al., 2009); CNR1 (Rempel et al., 2013); CNR2 (Rempel et al., 2013); GABA_A_ (Taferner et al., 2011); RXR (Kotani et al., 2010)	CYP19A1;ESR2;HSD17B2;HSD17B1;ESR1;SLC22A6;MIF
ZS	Naringin	CYP19A1 (Endringer et al., 2008)	ACHE;SLC5A4;AKR1B1;SLC5A2;ADORA1;CA14;CA12;SLC5A1;CA2;CA9;CA1

*^*a*^Data extracted from BindingDB (https://www.bindingdb.org). ^*b*^Predictions were made by using MOST to search against the pK_i_/pIC_*50*_/pEC_**50**_ dataset from CHEMBL20. Positive bioactivity data was defined as pIC_*50*_/pEC_*50*_/pKi > 6, while negative data was defined as pIC_*50*_/pEC_*50*_/pKi ≤ 6.*

Secondly, a computational method, namely MOST (Huang et al., 2017), was used to predict the possible targets of representative compounds. The machine learning models of MOST were trained by datasets from CHEMBL19 database (Gaulton et al., 2012). The prediction accuracies for pIC_50_, pEC_50_, and pK_i_ datasets are 73.5, 80.4, and 75.5%, respectively in temporal validation, where pIC_50_/pEC_50_/pK_i_ ≥ 6 are defined as “active” (Supplementary Table S8). Then, the representative compounds were searched against the datasets from CHEMBL20 (Supplementary Table S9) and corresponding predictions were made. In general, 14 predicted targets for emodin, 10 predicted targets for amygdalin, 7 predicted targets for albiflorin, 7 predicted targets for honokiol, and 11 predicted targets for naringin were found (**Table 1**).

Next, we checked which biological targets (from both referenced and predicted targets) of MZRW representative compounds have direct links with the symptom of constipation. By searching with PubMed, seven targets have been reported to be associated with constipation (**Table 2**).

**Table 2:** Referenced and predicted targets correlated with constipation.

Target	Full name	Linked compounds^a^	Links with constipation
ACHE	Acetylcholinesterase	Amygdalin; Naringin	The grade of ACHE expression in GI tissues correlates with eventual outcome and surgery requirement in patients with refractory constipation. More aggressive therapies are needed by patients with high-grade ACHE-positive distribution (Kobayashi et al., 2002). Acotiamide, a selective inhibitor of ACHE, was shown to stimulate GI motor activity in conscious dogs and approved for treatment of patients with functional dyspepsia (Nagahama et al., 2012; Nolan and Scott, 2013).
ADORA1	Adenosine A1 receptor	Naringin	Highly selective A1 and A2A agonists induce constipation in rats (Coupar and Tran, 2002).
CNR1	Cannabinoid receptor 1	**Honokiol**	Activation of CNR1 receptors inhibits the transmission of excitatory enteric neurons, leading to reduction of motility (Aviello et al., 2008). The inverse agonist of CNR1, taranabant, increased intestinal transit and reduced abdominal pain in mice (Fichna et al., 2013).
CYP19A1	cytochrome P450, family 19, subfamily A, polypeptide 1 (aromatase)	Honokiol; **Naringin**	Aromatase is a key enzyme for the biosynthesis of estrogens (Czajka-Oraniec and Simpson, 2010). Estrogen cause constipation in both female and male mice (Oh et al., 2013).
ESR2	estrogen receptor 2	**Emodin**; Honokiol	ESR2 is expressed in rectal samples of all controls, and decreased in obstructed defecation patients enteric neurons and glial cells (Bassotti et al., 2012). ESR2 is expressed in surface epithelial cells of proximal colon, and increased significantly during pregnancy in rats. ESR2 is implicated in constipation in pregnancy (Choijookhuu et al., 2012).
PTGS1	prostaglandin H2 synthase 1	**Honokiol**	Patients with slow transit constipation (STC) have lower PTGS1 protein and mRNA than controls (Cong et al., 2007).
PTGS2	prostaglandin H2 synthase 2	**Honokiol**	Patients with STC have higher PTGS2 protein and mRNA than controls (Cong et al., 2007).

*^*a*^Compound name in bold represents that compound-target link are supported by reference.*

**FIGURE 3:**
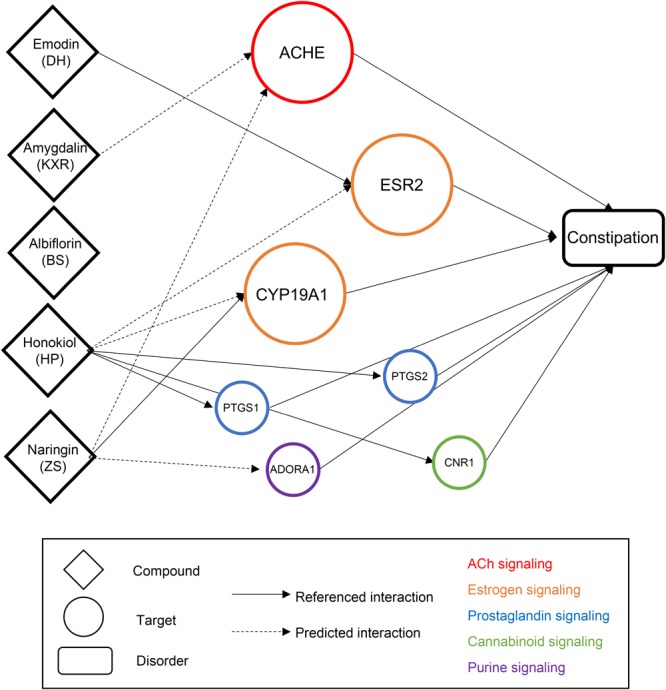
The drug-target-disorder network of MZRW representative compounds for constipation. The prokinetic effects of MZRW representative compounds can be explained by several targets. The compound is linked with biological target if it has reported (solid line) or predicted (dashed line) to be interacted with that target. All the biological targets have been reported to have links with constipation. Overall, these biological targets are belonging to the ACh signaling (red), estrogen signaling (orange), prostaglandin signaling (blue), cannabinoid signaling (green), and purine signaling (purple) pathways.

Finally, we generated the component group-target-disorder network of MZRW for FC (**Figure 3**). Albiflorin was not connected into this network since no targets of albiflorin have been reported to be linked with constipation. For the remaining four compounds, emodin, amygdalin, honokiol, and naringin, they were linked with constipation through seven targets, which can be categorized into five signaling pathways: acetylcholine (ACh), estrogen, prostaglandin, cannabinoid, and purine. All of these signaling pathways are related with constipation or gastrointestinal (GI) motility.

## Discussion

To investigate the pharmacology of TCM formula is a daunting task because of the complicated constituents and mechanism-of-actions. Network pharmacology was thought to be an important step to TCM modernization and a new research paradigm for the translation of TCM into evidence-based-medicine system (Liu and Sun, 2012; Li and Zhang, 2013). To date, more than 100 articles have been published in the cross-section of Chinese Medicine and network pharmacology. However, with this classic approach, typically hundreds of compounds are mapped to hundreds/thousands of targets, which finally would generate too many hypotheses to be tested. In our work studying the pharmacology of MZRW for FC, we used an improved strategy, namely, focused network pharmacology. Instead of looking for all the herbal compounds from public database and literatures, only compounds in the extracts that can be validated by quantitative method (UPLC-QTOF-MS/MS), were included. Such improvement provide a ‘real chemical space’ for subsequent analysis. We also proposed a hierarchical representation, herb-component group-representative compound, to abstract the chemical space. Based on the hierarchical clustering analysis, MZRW were seen as combination of several component groups, while each component group was represented by a major compound: that is the reason we named it “focused.” Compared with the traditional network pharmacology approach, the focused network pharmacology approach generate feasible number of mechanistic hypotheses, which are much easier to be tested experimentally. Very recently, several groups also used similar strategy to investigate the mechanism-of-actions of TCM. For example, Gao et al. (2018) proposed a new idea, “main active compound-based network pharmacology” to investigate the anti-cancer mechanism of Compound Kushen Injection. Four main active compounds were confirmed by UPLC-MS and cell proliferation assay, and the potential targets and pathways related with anti-HCC effects were predicted by network pharmacology approach. In another study performed by Zhao and He (2018), the main active compounds of Ganoderma lucidum extract were identified by HPLC, EI-MS, and NMR, and related targets were predicted by reverse-docking. Taken together, the focused network pharmacology approach could suggest a new direction in TCM pharmacology study: from explainable science to testable science.

In TCM theory, MZRW can drain heat, unblock the bowel, promote the movement of Qi, and moisten the intestines (Cheng et al., 2011). With modern pharmacology study, it was found that administration of MZRW increase the fecal pellet number and weight in mice. MZRW also enhance the contractions of intestinal segments of rabbit and guinea pig (ref?). Our work identified MZRW active components enhancing the colonic motility and analyzed the potential targets and pathways. It is an important step to understand the mechanisms of MZRW for FC at the molecular levels. It will also promote the quality control and modernization of MZRW.

Functional constipation is a highly heterogeneous disorders: psychological and psychiatric problems, personality traits, neuroendocrine and neurological changes, have been suggested to contribute to the development of slow transit constipation (STC), a major subtype of FC (Surrenti et al., 1995; Velio and Bassotti, 1996; El-Salhy, 2003; Frattini and Nogueras, 2008; Shahid et al., 2012). We found that several component groups (DH and ZS) could improve the constipation symptoms through modulating the estrogen signaling pathway. Naringin from ZS and Emodin from DH, through acting on estrogen production (CYP19A1) and estrogen-induced transcriptional activation (ESR2), cooperatively inhibit the estrogen signaling pathway (**Figure 3**). It has been found that FC is about two-times prevalent in women [17.4% (95%CI: 13.4–21.8%)] than in men [9.2% (95%CI: 6.5–12.2%)] (Suares and Ford, 2011). Consistently, progesterone and estrogen are implicated in the development of constipation (Chen et al., 1995; Knowles et al., 2003; Xiao et al., 2005, 2009; Oh et al., 2013). Since none of estrogen therapy has been approved for FC, the lesson from MZRW provide the clues to develop such novel therapies.

Our work also have several limitations. Firstly, due to the technical problems, fatty acids (mainly from HMR) were excluded from organ bath test and network analysis. Traditionally, the fatty acids are thought to have lubricant effects in treating constipation. However, there are increasing evidences suggest that the fatty acids may also have pharmacological effects in increasing bowel movement. For example, ricinoleic acid, major compound from castor oil, was found to induce laxation and uterus contraction by activating the EP_3_ receptors (Tunaru et al., 2012). Thus, it will be interested to investigate the pharmacological actions of fatty acid compounds of MZRW in the future. Secondly, the representative compounds of MZRW were identified but the combinational effect of them, have not been investigate yet. It will be of great research interest, to test if they have synergistic, additive, or antagonistic effect in future studies. Thirdly, several signaling pathways have been predicted by the focused network pharmacology approach, but the contribution of each pathway in enhancing colonic motility have not been measured. For individual animal subject, the contribution of single pathway could be quantitatively measured with highly specific pathway inhibition during the MZRW treatment. For the FC population, the capability of targeting multiple signaling pathway may offer MZRW the advantage over single-compound-agent in treating FC patients with different pathogenic causes.

## Conclusion

We developed a novel idea, namely “focused network pharmacology,” to investigate the pharmacological actions of MZRW for FC. Representative compounds of component groups in MZRW, emodin from DH, amygdalin from KXR, albiflorin from BS, honokiol from HP, and naringin from ZS induce spontaneous contractions of rat colonic smooth muscle *in vitro*. Biological targets of the representative compounds are within ACh-, estrogen-, prostaglandin-, cannabinoid-, and purine signaling pathways, which explain the prokinetic effects of corresponding component groups and herbs. MZRW active components enhance colonic motility, possibly by acting on multiple targets and pathways.

## Author Contributions

ZB and TH designed the whole study. TH carried out the chemical space analysis and target analysis. DH and ZN performed compound identification experiments. MZ and ZN did animal testing with the help from LZ and CL. ZB and TH analyzed the data and wrote the manuscript. LLDZ, HX, and ZY made contributions to the manuscript preparation.

## Conflict of Interest Statement

The authors declare that the research was conducted in the absence of any commercial or financial relationships that could be construed as a potential conflict of interest.

## Footnotes

1 http://bioinfogp.cnb.csic.es/tools/venny/

2 RDKit: Open-source cheminformatics: http://www.rdkit.org

3 https://www.bindingdb.org

4 https://www.ncbi.nlm.nih.gov/pubmed/
