# Supplementary material for: Uncovering the Mechanisms of Chinese Herbal Medicine (MaZiRenWan) for Functional Constipation by Focused Network Pharmacology Approach
Source: Front Pharmacol. 2018 Mar 26;9:270. doi: 10.3389/fphar.2018.00270 (PMC5879454; doi:10.3389/fphar.2018.00270)
Supplement: Supplementary file 1 [file Table_1.DOCX]

**Table S1. Compounds in MZRW extracts, and plasma and feces of rats with oral administration of MZRW**

| **No.** | **Cmpd_Name** | **Herb Source^a^** | **In Extract** | **In Plasma** | **In Feces** |
| --- | --- | --- | --- | --- | --- |
| 1 | (-)-Epicatechin-3-O-gallate | DH | TRUE | TRUE | FALSE |
| 2 | (+)-catechin | DH;BS | TRUE | FALSE | FALSE |
| 3 | (+)-catechin-5-O-β-D-glucopyranoside | DH | TRUE | FALSE | FALSE |
| 4 | 1,2,3-benzenetriol | BS | TRUE | FALSE | FALSE |
| 5 | 1,6-di-O-galloylycerol-β-D-glucopyranoside | DH | TRUE | FALSE | FALSE |
| 6 | 1,8-Dihydroxyanthraquinone | DH | TRUE | TRUE | FALSE |
| 7 | 10R-chrysaloin1-O-β-D-glucopyranoside | DH | TRUE | FALSE | FALSE |
| 8 | 1-O-galloyl-6-O-cinnamoyl-β-D-glucose | DH | TRUE | FALSE | FALSE |
| 9 | 2-​Acetylemodin 8-​O-​β-​D-​glucoside | DH | TRUE | FALSE | FALSE |
| 10 | 2,5-dimethyl-7-hydroxychromone | DH | TRUE | TRUE | TRUE |
| 11 | 2-O-cinnamoyl-β-D-glucose | DH | TRUE | TRUE | FALSE |
| 12 | 2-O-p-coumaroyl-1-O-galloy-β-D-glucose | DH | TRUE | FALSE | FALSE |
| 13 | 4',5-dihydroxyflavanone-7-O-β-D-glucoside | BS | TRUE | TRUE | TRUE |
| 14 | 4''-hydroxyl-albiflorin | BS | TRUE | FALSE | FALSE |
| 15 | 5,7-dihydroxy flavanone-4'-O-β-D-glucoside | BS | TRUE | FALSE | FALSE |
| 16 | 6-methyl-rhein | DH | TRUE | TRUE | TRUE |
| 17 | 6'-O-galloylalbiflorin | BS | TRUE | FALSE | FALSE |
| 18 | 6-O-galloylglucose | DH | TRUE | FALSE | FALSE |
| 19 | 6-O-galloylsucrose | DH | TRUE | FALSE | FALSE |
| 20 | Acteoside | HP | TRUE | FALSE | FALSE |
| 21 | Albiflorin | BS | TRUE | TRUE | FALSE |
| 22 | Aloe-emodin | DH | TRUE | TRUE | FALSE |
| 23 | Aloe-emodin-1-O-β-D-glucopyranoside | DH | TRUE | TRUE | FALSE |
| 24 | Aloesone-7-O-β-D-glucopyranoside | DH | TRUE | FALSE | FALSE |
| 25 | Amygdalin | KXR | TRUE | TRUE | FALSE |
| 26 | Apigenin | HMR;ZS | TRUE | TRUE | FALSE |
| 27 | Benzeneacetaldehyde | HP | TRUE | TRUE | TRUE |
| 28 | Benzoic acid | BS | TRUE | TRUE | TRUE |
| 29 | Benzoylpaeoniflorin | BS | TRUE | FALSE | FALSE |
| 30 | Cannabisin A | HMR | TRUE | FALSE | FALSE |
| 31 | Cannabisin B | HMR | TRUE | FALSE | FALSE |
| 32 | Cascaroside C | DH | TRUE | FALSE | FALSE |
| 33 | Cascaroside D | DH | TRUE | FALSE | FALSE |
| 34 | Cassialoin | DH | TRUE | TRUE | TRUE |
| 35 | Chrysophanol | DH | TRUE | TRUE | FALSE |
| 36 | Chrysophanol-1-O-β-D-glucopyranoside | DH | TRUE | FALSE | FALSE |
| 37 | Chrysophanol-8-O-β-D-glucopyranoside | DH | TRUE | FALSE | FALSE |
| 38 | Citreorosein | DH | TRUE | TRUE | FALSE |
| 39 | Citrusin B | ZS | TRUE | FALSE | FALSE |
| 40 | Desoxyrhaponticin | DH | TRUE | FALSE | FALSE |
| 41 | Desoxyrhapontigenin | DH | TRUE | TRUE | FALSE |
| 42 | Emodin | DH | TRUE | TRUE | FALSE |
| 43 | Emodin-8-O-β-D-glucopyranoside | DH | TRUE | FALSE | FALSE |
| 44 | Epicatechin | DH | TRUE | FALSE | FALSE |
| 45 | Eriodictyol | ZS | TRUE | FALSE | FALSE |
| 46 | Gallic acid | DH;BS | TRUE | FALSE | FALSE |
| 47 | Gallic acid 3-O-β-D-glucopyranoside | DH | TRUE | FALSE | FALSE |
| 48 | Gallic acid 4-O-β-D-glucopyranoside | DH | TRUE | FALSE | FALSE |
| 49 | Glucogallin | BS | TRUE | FALSE | FALSE |
| 50 | Hesperetin | ZS | TRUE | FALSE | FALSE |
| 51 | Hesperetin-7-O-β-D-glucoside | ZS | TRUE | FALSE | FALSE |
| 52 | Hesperidin | ZS | TRUE | TRUE | FALSE |
| 53 | Honokiol | HP | TRUE | TRUE | FALSE |
| 54 | Honokitriol; (7R*,8R*)-form | HP | TRUE | TRUE | TRUE |
| 55 | Imperatorin | ZS | TRUE | TRUE | FALSE |
| 56 | Inositol | KXR | TRUE | TRUE | TRUE |
| 57 | Isomerazin | ZS | TRUE | FALSE | FALSE |
| 58 | Kaempferol | DH | TRUE | FALSE | FALSE |
| 59 | Kaempferol-3-O-rhamnoside | DH | TRUE | FALSE | FALSE |
| 60 | laccaic acid D | DH | TRUE | TRUE | FALSE |
| 61 | Limonin | ZS | TRUE | FALSE | FALSE |
| 62 | Lindleyin | DH | TRUE | FALSE | FALSE |
| 63 | Linoleic acid | HMR | TRUE | TRUE | FALSE |
| 64 | Magnaldehyde D | HP | TRUE | FALSE | FALSE |
| 65 | Magnatriol B | HP | TRUE | FALSE | FALSE |
| 66 | Magnolignan A | HP | TRUE | TRUE | FALSE |
| 67 | Magnolignan B; 7-Deoxy | HP | TRUE | FALSE | FALSE |
| 68 | Magnolol | HP | TRUE | TRUE | FALSE |
| 69 | Melitidin | ZS | TRUE | FALSE | FALSE |
| 70 | Mudanpioside B | BS | TRUE | FALSE | FALSE |
| 71 | Mudanpioside E | BS | TRUE | TRUE | FALSE |
| 72 | Mudanpioside I | BS | TRUE | FALSE | FALSE |
| 73 | Naringenin | ZS | TRUE | FALSE | TRUE |
| 74 | Naringin | ZS | TRUE | TRUE | FALSE |
| 75 | Neohesperidin | ZS | TRUE | FALSE | FALSE |
| 76 | Neoponcirin | ZS | TRUE | FALSE | FALSE |
| 77 | N-trans-caffeoyltyramine | HMR | TRUE | FALSE | FALSE |
| 78 | Obovatol | HP | TRUE | FALSE | FALSE |
| 79 | Oleic acid | HMR;KXR | TRUE | TRUE | FALSE |
| 80 | Paeoniflorin | BS | TRUE | TRUE | FALSE |
| 81 | Paeonin A | BS | TRUE | FALSE | FALSE |
| 82 | Physcion-1-O-β-D-glucopyranoside | DH | TRUE | FALSE | FALSE |
| 83 | Physcion-8-O-β-D-gentiobioside | DH | TRUE | FALSE | FALSE |
| 84 | Physcion-8-O-β-D-glucopyranoside | DH | TRUE | FALSE | FALSE |
| 85 | Piceatannol | DH | TRUE | FALSE | FALSE |
| 86 | Piceatannol 3-O-β-D-glucopyranoside | DH | TRUE | FALSE | FALSE |
| 87 | Poncirin | ZS | TRUE | FALSE | FALSE |
| 88 | Procyanidin B-1 | DH | TRUE | FALSE | FALSE |
| 89 | Procyanidin B-1-3-O-gallate | DH | TRUE | FALSE | FALSE |
| 90 | Prunasin | KXR | TRUE | TRUE | FALSE |
| 91 | Rhein | DH | TRUE | TRUE | TRUE |
| 92 | Rhein-8-O-β-D-glucopyranoside | DH | TRUE | TRUE | FALSE |
| 93 | Sativic acid | HMR | TRUE | FALSE | FALSE |
| 94 | Sennoside A | DH | TRUE | FALSE | FALSE |
| 95 | Sennoside B | DH | TRUE | FALSE | FALSE |
| 96 | Sennoside C | DH | TRUE | FALSE | FALSE |
| 97 | Synephrine | ZS | TRUE | FALSE | FALSE |
| ^a^HMR, *Huo Ma Ren* (*Fructus cannabis*); DH, *Da Huang* (*Radix et rihizoma rhei*); KXR, *Ku Xing Ren* (*Semen Armeniacae Amarum*); BS, *Bai Shao* (*Radix paeoniae Albo*); HP, *Hou Pu* (*Cortex magnolia officinalis*); ZS, *Zhi Shi* (*Fructus aurantll immaturus*). | | | | | |
