# Supplementary material for: Uncovering the Mechanisms of Chinese Herbal Medicine (MaZiRenWan) for Functional Constipation by Focused Network Pharmacology Approach
Source: Front Pharmacol. 2018 Mar 26;9:270. doi: 10.3389/fphar.2018.00270 (PMC5879454; doi:10.3389/fphar.2018.00270)
Supplement: Supplementary file 5 [file Table_5.DOCX]

**Table S5. Compounds of component group 4**

| **ID** | **Compound Name** | **Herb Source^a^** | **Structure** |
| --- | --- | --- | --- |
| 53 | Honokiol | HP | 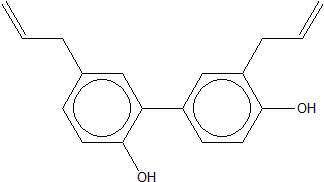 |
| 54 | Honokitriol; (7R*,8R*)-form | HP | 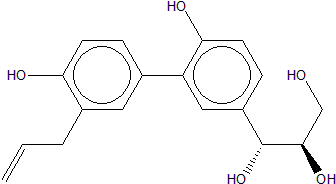 |
| 64 | Magnaldehyde D | HP | 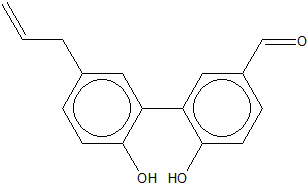 |
| 65 | Magnatriol B | HP | 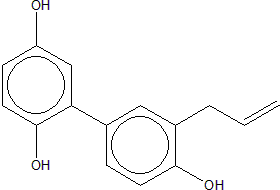 |
| 66 | Magnolignan A | HP | 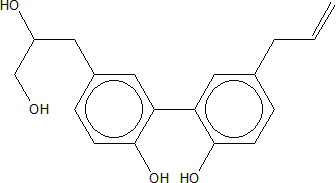 |
| 67 | Magnolignan B; 7-Deoxy | HP | 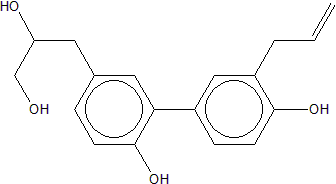 |
| 68 | Magnolol | HP | 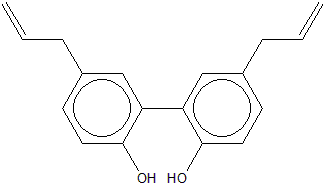 |
| 78 | Obovatol | HP | 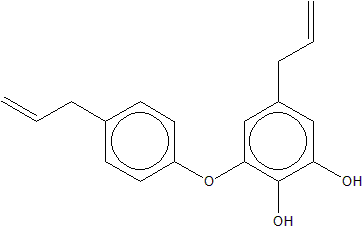 |
| ^a^HMR, *Huo Ma Ren* (*Fructus cannabis*); DH, *Da Huang* (*Radix et rihizoma rhei*); KXR, *Ku Xing Ren* (*Semen Armeniacae Amarum*); BS, *Bai Shao* (*Radix paeoniae Albo*); HP, *Hou Pu* (*Cortex magnolia officinalis*); ZS, *Zhi Shi* (*Fructus aurantll immaturus*). | | | |
