# Supplementary material for: Uncovering the Mechanisms of Chinese Herbal Medicine (MaZiRenWan) for Functional Constipation by Focused Network Pharmacology Approach
Source: Front Pharmacol. 2018 Mar 26;9:270. doi: 10.3389/fphar.2018.00270 (PMC5879454; doi:10.3389/fphar.2018.00270)
Supplement: Supplementary file 7 [file Table_7.DOCX]

**Table S7. Referenced targets of MZRW main compounds^a^**

| **Herb** | **Compound** | **# of referenced targets** | **Target** | **Bioactivity data** |
| --- | --- | --- | --- | --- |
| DH | Emodin | 7 | ESR1 (ER_α_) | K_i_ = 0.77 μM^[1](#_ENREF_1)^ |
|  |  |  | ESR2 (ER_β_) | K_i_ = 1.5 μM^[1](#_ENREF_1)^ |
|  |  |  | CSNK2A1 (CK2) | IC_50_ = 0.89 μM^[2](#_ENREF_2)^;  K_i_ = 1.85 μM^[2](#_ENREF_2)^ |
|  |  |  | EPHX2 (sEH) | IC_50_ = 36.4 μM^[3](#_ENREF_3)^ |
|  |  |  | PTP4A3 (PRL-3) | IC_50_ = 3.5 μM^[4](#_ENREF_4)^ |
|  |  |  | LCK | IC_50_ = 18.5 μM^[5](#_ENREF_5)^ |
|  |  |  | ELANE (HLE) | IC_50_ = 52 μM^[6](#_ENREF_6)^ |
| HP | Honokiol | 7 | PTGS1 (COX-1) | IC_50_ = 1.8 μM^[7](#_ENREF_7)^ |
|  |  |  | PTGS2 (COX-2) | IC_50_ = 2.1 μM^[7](#_ENREF_7)^ |
|  |  |  | ALOX5 (5-LOX) | IC_50_ = 4.2 μM^[7](#_ENREF_7)^ |
|  |  |  | CNR1 (CB1) | Ki = 6.46 μM^[8](#_ENREF_8)^ |
|  |  |  | CNR2 (CB2) | Ki = 5.61 μM^[8](#_ENREF_8)^ |
|  |  |  | GABA_A_ | EC_50_ ~ 23.4 to 59.6 μM^[9](#_ENREF_9)^ |
|  |  |  | RXR | EC_50_ = 11.8 μM^[10](#_ENREF_10)^ |
| ZS | Naringin | 1 | CYP19A1 | IC_50_ = 5.0 μM^[11](#_ENREF_11)^ |
| ^a^Data extracted from BindingDB (<https://www.bindingdb.org>) followed by manual curation. | | | | |

# Reference

1. Matsuda, H., Shimoda, H., Morikawa, T. & Yoshikawa, M. Phytoestrogens from the roots of Polygonum cuspidatum (Polygonaceae): structure-requirement of hydroxyanthraquinones for estrogenic activity. *Bioorg Med Chem Lett* **11**, 1839-42 (2001).

2. Sarno, S. et al. Biochemical and three-dimensional-structural study of the specific inhibition of protein kinase CK2 by [5-oxo-5,6-dihydroindolo-(1,2-a)quinazolin-7-yl]acetic acid (IQA). *Biochem J* **374**, 639-46 (2003).

3. Lee, G.Y., Kim, J.H., Choi, S.K. & Kim, Y.H. Constituents of the seeds of Cassia tora with inhibitory activity on soluble expoxide hydrolease. *Bioorg Med Chem Lett* **25**, 5097-101 (2015).

4. Han, Y.M. et al. Emodin inhibits migration and invasion of DLD-1 (PRL-3) cells via inhibition of PRL-3 phosphatase activity. *Bioorg Med Chem Lett* **22**, 323-6 (2012).

5. Chang, C.J. & Geahlen, R.L. Protein-tyrosine kinase inhibition: mechanism-based discovery of antitumor agents. *J Nat Prod* **55**, 1529-60 (1992).

6. Zembower, D.E., Kam, C.M., Powers, J.C. & Zalkow, L.H. Novel anthraquinone inhibitors of human leukocyte elastase and cathepsin G. *J Med Chem* **35**, 1597-605 (1992).

7. Schuhly, W. et al. Design and synthesis of ten biphenyl-neolignan derivatives and their in vitro inhibitory potency against cyclooxygenase-1/2 activity and 5-lipoxygenase-mediated LTB4-formation. *Bioorg Med Chem* **17**, 4459-65 (2009).

8. Rempel, V. et al. Magnolia Extract, Magnolol, and Metabolites: Activation of Cannabinoid CB2 Receptors and Blockade of the Related GPR55. *Acs Medicinal Chemistry Letters* **4**, 41-45 (2013).

9. Taferner, B. et al. Modulation of GABAA-receptors by honokiol and derivatives: subtype selectivity and structure-activity relationship. *J Med Chem* **54**, 5349-61 (2011).

10. Kotani, H., Tanabe, H., Mizukami, H., Makishima, M. & Inoue, M. Identification of a Naturally Occurring Rexinoid, Honokiol, That Activates the Retinoid X Receptor. *Journal of Natural Products* **73**, 1332-1336 (2010).

11. Endringer, D.C., Guimaraes, K.G., Kondratyuk, T.P., Pezzuto, J.M. & Braga, F.C. Selective inhibition of aromatase by a dihydroisocoumarin from Xyris pterygoblephara. *J Nat Prod* **71**, 1082-4 (2008).
