# Supplementary material for: Uncovering the Mechanisms of Chinese Herbal Medicine (MaZiRenWan) for Functional Constipation by Focused Network Pharmacology Approach
Source: Front Pharmacol. 2018 Mar 26;9:270. doi: 10.3389/fphar.2018.00270 (PMC5879454; doi:10.3389/fphar.2018.00270)
Supplement: Supplementary file 8 [file Table_8.DOCX]

**Table S8. Overall performance of MOST in temporal validation**

| **Dataset** | **pIC_50_** | **pEC_50_** | **pK_i_** |
| --- | --- | --- | --- |
| # of targets | 410 | 79 | 173 |
| # of total bioactivity data in training set^a^ | 109,022  (positive: 69,028; negative: 39,994)^b^ | 15,762  (positive: 11,853; negative: 3,909) | 61,937  (positive: 46,875; negative:15,062) |
| # of total bioactivity data in testing set^c^ | 9,338  (positive: 5407; negative: 3,931) | 1,291  (positive: 1,005; negative: 286) | 3,754  (positive: 2,757; negative: 997) |
| Accuracy | 73.5% | 80.4% | 75.5% |
| MCC | 0.471 | 0.386 | 0.382 |
| False positive rate | 19.8% | 14.9% | 16.1% |
| False negative rate | 33.7% | 32.9% | 46.2% |
| ^a^The training set was generated from CHEMBL19.  ^b^Positive bioactivity data was defined as pIC_50_/pEC_50_/pKi>6, while negative data was defined as pIC_50_/pEC_50_/pKi≦6.  ^c^The testing set was generated from the newly deposited ligands in CHEMBL20 compared with CHEMBL19. | | | |
