# Supplementary material for: Uncovering the Mechanisms of Chinese Herbal Medicine (MaZiRenWan) for Functional Constipation by Focused Network Pharmacology Approach
Source: Front Pharmacol. 2018 Mar 26;9:270. doi: 10.3389/fphar.2018.00270 (PMC5879454; doi:10.3389/fphar.2018.00270)
Supplement: Supplementary file 9 [file Table_9.DOCX]

**Table S9. Statistics of CHEMBL 20 pIC_50_/pEC_50_/pK_i_ dataset for searching**

| **Dataset** | **pIC_50_** | **pEC_50_** | **pK_i_** |
| --- | --- | --- | --- |
| # of targets | 1,300 | 364 | 725 |
| # of total bioactivity data | 148,539  (positive: 94,124; negative: 54,415)^a^ | 28,347  (positive: 20,717; negative: 7,630) | 84,254  (positive: 63,237; negative:27,017) |
| ^a^Positive bioactivity data was defined as pIC_50_/pEC_50_/pKi>6, while negative data was defined as pIC_50_/pEC_50_/pKi≦6. | | | |
